# Re-emergence memory of subtropical mode-water links Atlantic and Pacific multidecadal variability

**DOI:** 10.1093/nsr/nwaf047

**Published:** 2025-02-14

**Authors:** Baolan Wu, Xiaopei Lin, Lisan Yu

**Affiliations:** Advanced Institute for Marine Ecosystem Change (WPI-AIMEC), Tohoku University, Sendai 980-0845, Japan; Frontier Science Center for Deep Ocean Multispheres and Earth System and Physical Oceanography Laboratory, Ocean University of China, Qingdao 266100, China; Laboratory for Ocean Dynamics and Climate, Qingdao Marine Science and Technology Center, Qingdao 266100, China; Department of Physical Oceanography, Woods Hole Oceanographic Institution; Woods Hole, MA 02543, USA

**Keywords:** North Pacific Subtropical Mode Water, Kuroshio–Oyashio Extension, ocean multidecadal variability, ocean recirculation, re-emergence

## Abstract

Understanding how the ocean provides the memory for maintaining decadal to multidecadal variability is key to climate prediction. An important question is what process could provide the observed significant time lag between the Pacific Decadal Oscillation (PDO) and the Atlantic Multidecadal Oscillation (AMO). Here, we show that the Pacific Ocean could store climate anomalies within the subtropical recirculation gyre for a decadal duration, providing a ‘seeding’ link between the AMO and the PDO. The AMO-induced multidecadal temperature anomalies in the Northwestern Pacific Ocean are subducted into the thermocline and sequestered in the North Pacific Subtropical Mode Water. These temperature anomalies propagate along the recirculation gyre and eventually rejoin the Kuroshio one decade later. Once re-emerged in the Kuroshio–Oyashio Extension region, the anomalies may engage with local air–sea feedback, triggering the PDO. Our findings demonstrate that the mode water could provide a much-needed ocean memory for the prediction of multidecadal variability.

## Teaser

Pacific Ocean circulation could store and transport the Atlantic Ocean climate anomalies in the subsurface water mass for a decade-long time. This is the key to link the Atlantic and Pacific Ocean decadal variability.

## INTRODUCTION

Understanding the cause of decadal and multidecadal ocean variability, such as the Pacific Decadal Oscillation (PDO) [1] and the Atlantic Multidecadal Oscillation (AMO) [2], is crucial for the prediction of large-scale climate variability and change [3]. Several mechanisms have been proposed to explain the memory that supports interannual to decadal variability in the North Pacific Ocean, including the ocean heat transport oscillation that is sustained by positive ocean–atmosphere feedback in the Kuroshio–Oyashio Extension (KOE) [4,5], the remote tropical forcing that is transmitted through both the atmospheric and oceanic bridges [6], the slowly propagating oceanic Rossby waves that are excited by stochastic wind stress forcing [7] and the decadal changes in the KOE that are driven by wind-forced oceanic Rossby waves [8]. On multidecadal timescales, studies have shown that the AMO and the PDO are connected [9], with the AMO leading the PDO by ∼12 years [10–12].

Central to the explanation of the AMO and the PDO connection is how the AMO-induced temperature anomalies are sustained for a decade before triggering a PDO. Recent studies found that the sea surface temperature (SST) anomalies that are generated by an AMO are sequestered in the subsurface Northwestern Pacific Ocean with nearly no time lag [13,14]. Previous studies have suggested that subsurface temperature anomalies in the mid-latitude Pacific Ocean could propagate along the interior pathway toward the tropical Pacific Ocean and are then resurfaced by the equatorial upwelling about one decade later [6,15]. By interacting with local atmosphere, the re-emerged temperature anomalies generate a PDO-like SST pattern through the Pacific–North American teleconnection [16]. This pathway appears to be a viable ocean memory for the PDO–AMO interaction on decadal timescales. However, the presence of a potential vorticity barrier at about 10^o^N basically prevents this pathway from reconnecting with the formation region of the anomalies [17]. Other studies considered a local re-emergence mechanism [18,19] in the KOE and explored the mid-latitude air–sea interaction by focusing on the potential effects of the SST anomalies that emerged from the subsurface ocean on the storm track, the Aleutian Low and the PDO [20].

However, the local re-emergence could only last for a number of years, so the way in which the temperature anomalies could excite a PDO on decadal lag times remains unknown. The ocean sustains the low-frequency climate variations through its huge heat content, which is usually provided by the subsurface ocean water mass such as the North Pacific Subtropical Mode Water (STMW; see ‘Definition of the STMW’ in ‘Methods’, Supplementary Data). Inspired by that, we further reveal that the decadal signal is stored in the STMW before being carried by the ocean recirculation and rejoining the surface Kuroshio Extension region one decade later to be a potential trigger of the PDO.

## RESULTS

### STMW propagation along the recirculation gyre links the PDO and the AMO

**Figure 1.**
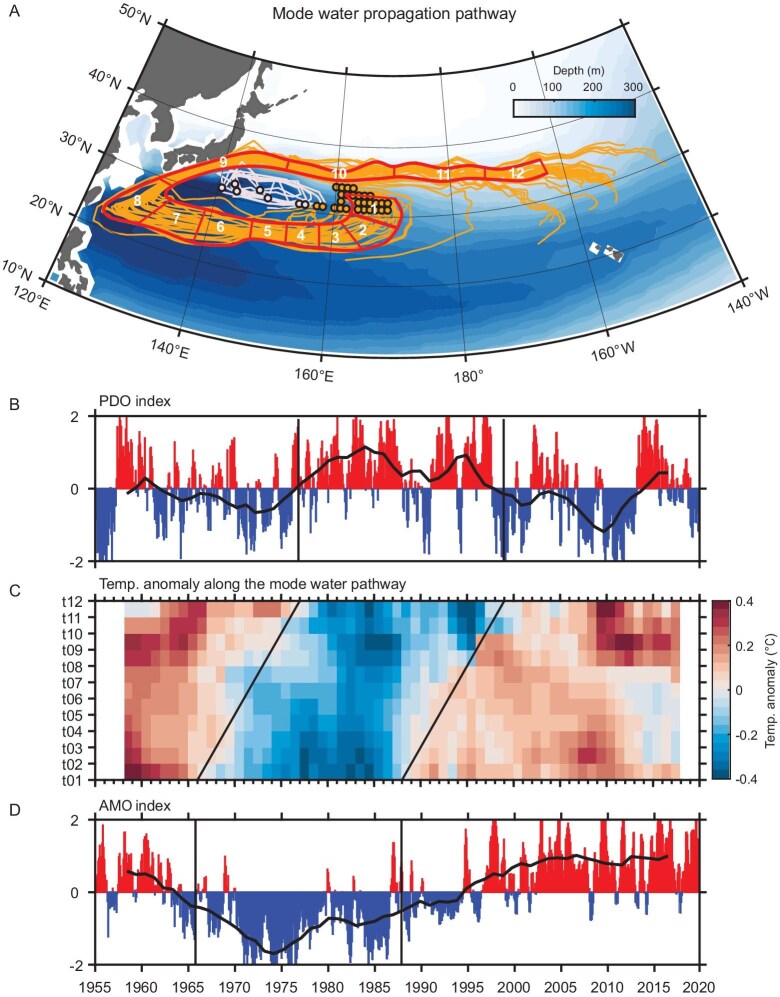
Time-lag-correlated PDO and AMO caused by the temperature anomalies that propagate along the STMW pathway. (A) Climatological pathway of the STMW for the isopycnal surfaces between 25.0 and 25.6 kg m^−3^ (shading, in m). Solid yellow and white lines represent the trajectories that were analysed by using a Lagrange-tracking method. Note that the trajectory is calculated by the STMW definition whose thickness is >50 m. The white dots indicate the releasing positions of the trajectories in the western STMW formation region, which are trapped locally and dissipated by local re-emergence processes. The yellow dots indicate the releasing positions of the trajectories in the eastern STMW formation region, which can propagate along the thermocline circulation and eventually re-emerge remotely at the KOE. The positions of the 12 sections along the pathway are marked, with each section representing the travel time of ∼1 year. (B) Normalized monthly PDO index and its 7-year low-pass filtered time series (solid line). (C) Seven-year low-pass filtered temperature anomalies (in °C) averaged between 25.0 and 25.6 kg m^−3^ isopycnal surfaces along the 12 sections (i.e. the *y*-axis t01–t12 denotes 1–12 sections shown in Fig. 1A). (D) Normalized monthly AMO index and its 7-year low-pass filtered time series (solid line). (A) and (C) are derived from the Ishii data.

Here, we show that the lagging of the PDO behind the AMO by one decade is controlled by the propagation time of the temperature anomalies that are carried by the STMW [21] along its interior pathway. To trace the propagation of the temperature anomalies that are carried by the STMW, we reconstruct the pathway of the water masses between the isopycnal surfaces of 25.0 and 25.6 kg m^−3^—the typical density range of the STMW [22], based on the Lagrangian method ([23,24]; see ‘Lagrange-tracking method’ in ‘Methods’, Supplementary Data). As shown in Fig. 1A, the STMW west of 160^o^E (white dots and lines) is trapped locally and cannot transport the temperature anomalies outside the formation region. The STMW that is formed east of 160^o^E (yellow dots and lines in Fig. 1A) is subducted into the thermocline, which rides on the recirculation gyre with low potential vorticity (Fig. S1) along the subtropical front ([25]; Fig. S2) and flows southwestward.

The subtropical front is key for maintaining the AMO-induced signal inside the thermocline by preventing strong dissipation of the local re-emergence process during the signal propagation. This body of water eventually joins the Kuroshio to flow northeastward and then turns towards the east at about 35^o^N along the KOE. The total distance of the recirculation pathway is ∼16 000 km and the averaged current speed in the thermocline between the isopycnal surfaces of 25.0 and 25.6 kg m^−3^ is ∼0.05 m s^−1^ (Fig. S1). Hence, it takes >10 years for the STMW to travel from its subduction site to the KOE area.

**Figure 2.**
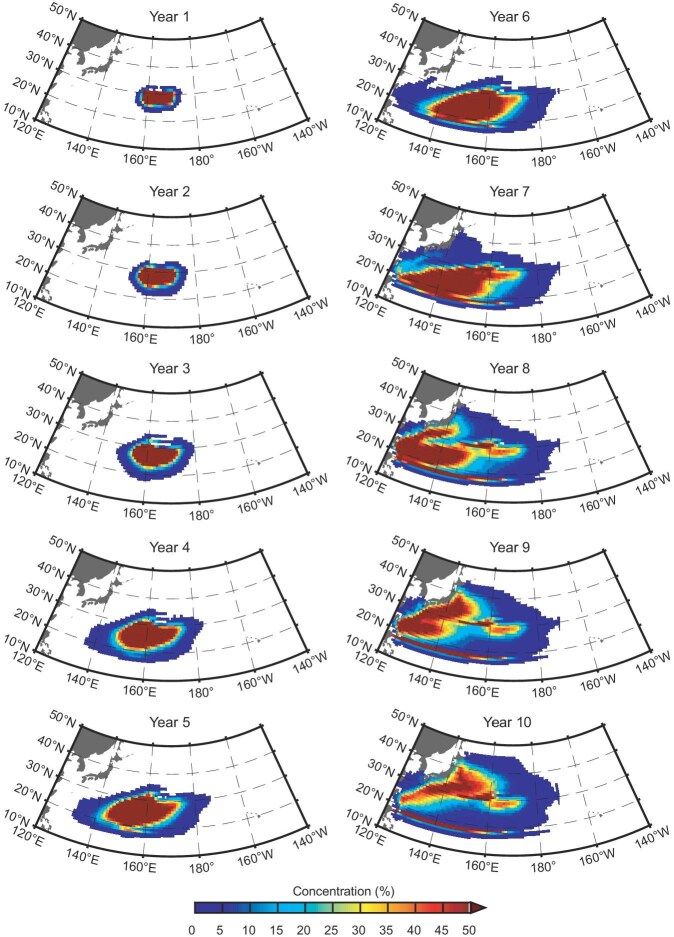
Spatial distribution of the passive tracer concentration along the STMW pathway. Year-to-year evolution of the passive tracer averaged in the STMW (shading, in %) from Years 1–10. The results are based on the passive tracer EXP. from the ECCO model (see ‘Methods’).

In addition to the observations, one data-assimilated model (Simple Ocean Data Assimilation (SODA) [26]; Fig. S3), a pre-industrial control simulation (200 years) and a pacemaker experiment by Community Earth System Model (CESM [27]; see ‘Pacemaker model experiment’ and ‘Pre-industrial model simulation experiment’ in ‘Methods’, Supplementary Data, and Figs S4–S6) with multiple AMO–PDO cycles support that the oceanic pathway of the STMW propagation determines the time lag that links the PDO to the AMO. Furthermore, an offline passive tracer from a model output of the Consortium for Estimating the Circulation and Climate of the Ocean (ECCO) is employed to track the movement of the STMW anomalies ([28,29]; see ‘Passive tracer experiment’ in ‘Methods’, Supplementary Data). It is shown that >40% of the STMW that is formed in the east of 160^o^E could reach the KOE area ∼10 years after propagating along the recirculation gyre (Fig. 2), which is in agreement with the duration of the observed temperature anomalies carried by the STMW (Fig. 1).

When the AMO-induced temperature anomalies that are transported by the recirculation gyre re-emerge in the KOE area, they outcrop to the surface by a shoaling thermocline (shading in Fig. 1A derived from the Ishii data and shading in Figs S5A and S6A from the CESM outputs) and generate SST anomalies. Subsequently, these SST anomalies interact with the atmosphere and produce a PDO-like SST pattern in ∼12 years of elapsed time (Fig. 1B–D and Fig. S3). For example, the cold temperature anomalies in the STMW formation region (Pathway portion 1, Fig. 1C) during the AMO negative phase in 1965–90 (Fig. 1D) propagated along the STMW pathway (Pathway portions 2–8, Fig. 1C) to the KOE (Pathway portions 9–12, Fig. 1C), where the cool anomalies triggered a corresponding PDO positive phase during 1977–2000 (Fig. 1B).

**Figure 3.**
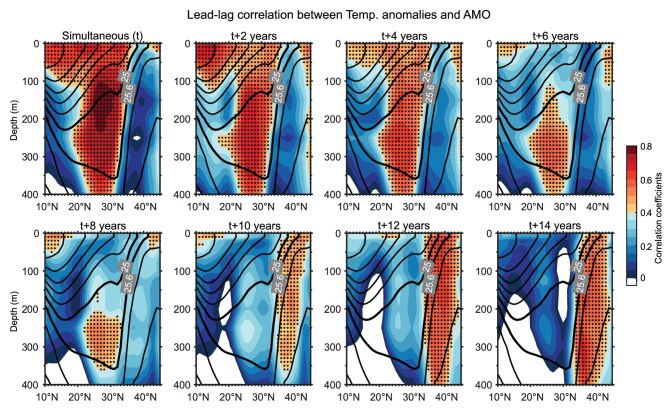
Vertical propagation of the temperature anomalies associated with subduction and re-emergence. The lead–lag correlation between temperature anomalies (averaged between 140°E and 170°E transect, derived from the Ishii data) and the AMO index, both smoothed using a 7-year low-pass filter, is shown at 2-year intervals (shading). Contours represent potential density with intervals of 0.6 kg m^−3^. Thick solid contours denote potential densities of 25.0 and 25.6 kg m^−3^, which define the upper and lower density boundaries of the STMW. Dot-shaded areas denote correlations of *t* significant at the 95% confidence interval.

To further understand the retention and re-emergence of the AMO-induced temperature anomalies by the STMW, we examine the related temperature variations in a vertical transect (Fig. 3). At lag 0 (*t*), the SST anomalies in the south of 35^o^N are generated by the AMO [13,14] through atmospheric teleconnections (e.g. stationary Rossby wave; [30]); these anomalies are then subducted into the thermocline in the wintertime when the mixed-layer depth reaches its maximum [13]. From Year 0 to Year 4 (Fig. 3, *t* to *t* + 4), the temperature anomalies are sequestered in the STMW. According to the existing local ocean re-emergence mechanism [18], these SST anomalies would persist in the subsurface layer through the summer and fall (Fig. S7) and resurface in the following winter (Fig. S8). As the re-emergence would last for only a few years [19] and diminish in Year 6 (Fig. 3, *t* + 6), the AMO-induced temperature anomalies in the locally trapped STMW (west of 160^o^E in Fig. 1A) would not be sustained on a decadal timescale. In our mechanism, the signal that is formed east of 160^o^E is embedded in the STMW, which is shielded from the direct atmospheric forcing during its travel along the subtropical circulation (Fig. 3, *t* + 6 and *t* + 8). In Year 10, these temperature anomalies reach the KOE region (Fig.3, *t* + 10) and resurface in the isopycnal outcropping area in the following several years (Fig. 3, *t* + 12 and *t* + 14).

### Mechanism differences between the remote and local re-emergence process

**Figure 4.**
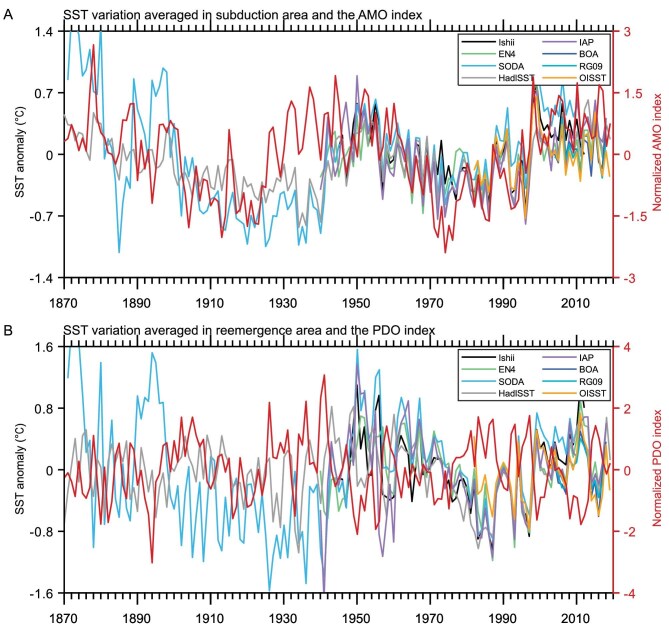
The relationship between region averaged SST anomalies, the AMO index and the PDO index with different data sources. (A) Time series of the SST variation (from eight kinds of data sources, as shown in the legend) averaged in the subduction area (130°–180°E, 25°–35°N) and the normalized AMO index (red line). (B) Time series of the SST variation (from eight kinds of data sources, as shown in the legend) averaged in the re-emergence area (140°–160°E, 35°–42°N) and the normalized PDO index (red line).

Our proposed re-emergence mechanism differs from the existing local re-emergence mechanism [18,19] in two respects. First, the temperature anomalies are sustained in the thermocline for >10 years, allowing the mechanism to be an ocean memory in explaining the 12-year time lag between the PDO and the AMO. Second, the re-emergence is not limited to the late winter, as suggested by the existing theory [18], but takes place all year round, as shown in Figs S7 and S8. This is further demonstrated in Fig. S9, which shows that the propagation of the temperature anomalies from its formation region to the mid-latitude is present in both winter and summer. When the AMO-induced SST anomalies (Fig. 4A) that are carried by the STMW arrive at the outcropping area, they re-emerge at the surface and exhibit multidecadal variability that is consistent with the PDO (Fig. 4B). The temperature anomalies spread further eastward into the entire KOE region (150°E–160°W, 35°–42°N), showing simultaneous multidecadal variation with the PDO and a 12-year lag with the AMO (Fig. S10).

**Figure 5.**
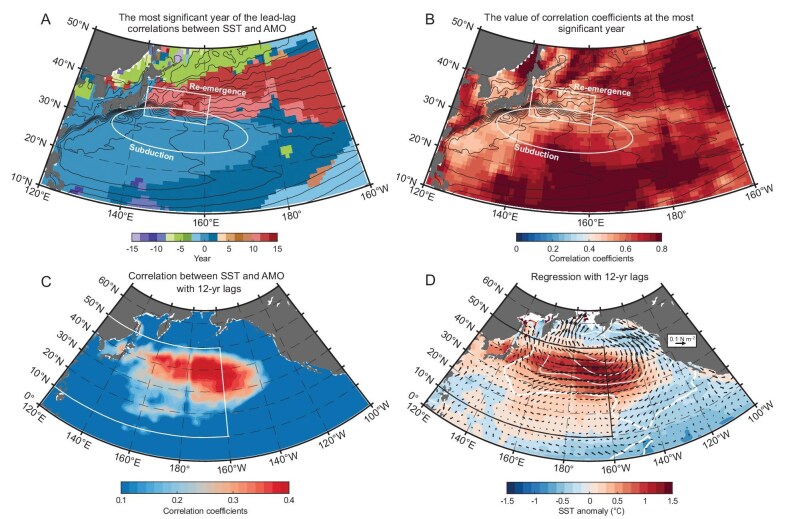
Re-emerged SST anomalies induced by the AMO may trigger a PDO after 12 years. The spatial structure of the year with (A) the strongest correlation and (B) its corresponding correlation coefficients, both smoothed using a 7-year low-pass filter (shading). Contours represent absolute dynamic topography derived from Archiving, Validation and Interpretation of Satellite Oceanographic data (AVISO), with a 10-cm interval. The white rectangle and oval denote the climatological re-emergence and subduction areas, respectively. (C) Spatial structure of correlations between the 7-year low-pass-filtered SST and the AMO index with a 12-year lag. (D) Time-lag regression of the SST (color shading, in °C), wind stress (vectors, in N/m^2^) and sea-level pressure (white contours, in hPa) on the AMO index at a 12-year lag. Contour intervals are 1.2 hPa, with positive contours shown as dashed lines and negative contours as solid lines. The thick white contour marks the 0-hPa sea-level pressure. The white and black boxes in (C) and (D) highlight the regions shown in (A) and (B).

The above ocean circulation re-emergence creates a pattern in the correlation between the North Pacific SST and the AMO. Figure 5A shows the spatial distribution of the times (in years) at which the lead–lag correlations between the AMO and the SST in the Northwest Pacific Ocean are highest and statistically significant, with Fig. 5B showing the value of the corresponding correlation coefficients. South of the Kuroshio Extension (contours in Fig. 5A), where a deepening of the mixed layer in later winter leads to a formation of the STMW [21], the SST varies simultaneously with the AMO (white oval and blue shading in Fig. 5A; [13,14]). On the other hand, the SST lags the AMO significantly by ∼12 years north of the Kuroshio Extension. The transition between the zero lag and the 12-year lag is sharp along the Kuroshio Extension in both winter (Fig. S11A) and summer (Fig. S11B), indicating that the SST anomalies on the two sides of the KE are influenced by the AMO signals one decade earlier.

Once they have re-emerged, these temperature anomalies can influence the front/eddy activities in the KOE region, potentially triggering a PDO-like pattern [31–33]. Observational evidence of this link is seen in the PDO pattern that is derived from correlations between the SST and the AMO with a 12-year lag (red shading in Fig. 5C), indicating the connection between the AMO and the PDO in the North Pacific Ocean. To further illustrate their connection, a sequence of time-lag regression maps of the SST, wind stress and sea-level pressure anomalies on the AMO index were computed at 9-, 10-, 11- and 12-year lags (Fig. S12 and Fig. 5D). The linkage between the AMO and the PDO is demonstrated through the evolution of variable distributions over time and space, as evidenced not only by observational data (Fig. 5D and Fig. S12; [10,12]), but also by the pacemaker experiment (Fig. S13). Furthermore, the resultant spatial patterns are consistent with anomaly patterns of the PDO (Fig. S14).

## DISCUSSION AND CONCLUSION

It should be noted that the contribution of mid-latitude air–sea interaction to the PDO remains unclear [16]. While some studies suggest that air–sea interaction in the KOE frontal region occurs at sub-meso to mesoscales (1–100 km) and in high-frequency (daily to monthly) scales [34,35], and may play a role in generating a PDO-like pattern through the atmospheric transient eddy forcing [32,33], recent studies indicate that SST anomalies in the KOE frontal region could only account for approximately one-third of the PDO variability [36]. Most of the PDO-like SST pattern in the North Pacific Ocean appears to be directly forced by changes in the Aleutian Low, which responds to SST anomalies in the eastern tropical Pacific Ocean. This is evident from the 9-year lag that persists to 12-year lag (Fig. S12). As the contribution of mid-latitude air–sea interaction remains unclear and subject to debate, further studies are necessary to determine the potential for SST anomalies that are transported by the STMW in the KOE region to generate a PDO in the future.

There have been considerable efforts made to explore the time lag in the ocean for sustaining decadal variability in the North Pacific Ocean, such as the first mode Oceanic Rossby Wave [10,37] and upper ocean advection [4]. However, the former provides an ocean memory for only a number of years and the latter mixes the first mode (Rossby waves) with higher-order (mode-water propagation) ocean variability and has yet to be identified from observations. Our findings demonstrate that the combined effects of horizontal advection (i.e. recirculation gyre) and vertical motion (i.e. subduction and re-emergence) play an important role in providing a decadal-long ocean memory. This new ocean circulation re-emergence allows the subducted temperature anomalies to be embedded in the STMW, propagate on the isopycnal surfaces and eventually resurface in the KOE region in ∼12 years. The mode-water recirculation pathway expands the concept of the local re-emergence [18,19,38,39].

In addition, a distinct difference exists between the PDO and the AMO: the PDO exhibits a pronounced ∼20-year oscillatory component alongside the ∼50-year oscillation [40,41] that covaries with the AMO. This ∼20-year oscillatory pattern of the PDO is driven by internal Pacific Ocean dynamics [16], indicating that the PDO displays considerable variability that is independent of the AMO. Although this topic is beyond the scope of this study, it warrants further investigation.

The air–sea interaction in the mid-latitudes is complex, making it challenging to find the predictable sources on decadal timescales. Recent studies have proposed that the AMO could be a potential predictor for the North Pacific [10–12] or even Eurasia climate change [42] with a lead time of a decade, but the mechanism is still unknown. Our study explains, for the first time, that the AMO-induced SST anomalies could be transmitted through the slow ocean circulation re-emergence mechanism for ∼12 years. This could provide a much-needed ocean memory for the prediction of multidecadal variability—a potential that should be seriously considered.

## METHODS

Detailed descriptions of methods are available in the Supplementary Data.

## Supplementary Material

nwaf047_Supplemental_File

## Data Availability

Data related to this paper can be downloaded from the following: Ishii data (version 7.3), https://climate.mri-jma.go.jp/; EN4 data (version 4.2.0-analyses-g10), https://www.metoffice.gov.uk/; IAP data, http://www.ocean.iap.ac.cn/pages/dataService/dataService.html; OISST data, https://www.esrl.noaa.gov/psd/data/gridded/data.noaa.oisst.v2.highres.html; HadlSST data, https://www.metoffice.gov.uk/hadobs/hadisst/data/download.html; RG09 data, http://www.argo.ucsd.edu/Gridded_fields.html; BOA data, ftp://data.argo.org.cn/pub/ARGO/BOA_Argo/; SODA data, https://climatedataguide.ucar.edu/climate-data/soda-simple-ocean-data-assimilation NCEP data, https://psl.noaa.gov/data/gridded/data.ncep.reanalysis.html;https://www.esrl.noaa.gov/psd/; ADT data, https://www.aviso.altimetry.fr/en/home.html; the PDO index, https://www.ncei.noaa.gov/access/monitoring/pdo/; the AMO index, https://climatedataguide.ucar.edu/climate-data/atlantic-multi-decadal-oscillation-amo. The Matlab is used for plotting.

## References

[1] Mantua NJ, Hare SR, Zhang Y et al. A Pacific interdecadal climate oscillation with impacts on salmon production. Bull Am Meteorol Soc 1997; 78: 1069–79. 10.1175/1520-0477(1997)078<1069:APICOW>2.0.CO;2

[2] Enfield DB, Mestas-Nunez AM, Trimble PJ. The Atlantic multidecadal oscillation and its relation to rainfall and river flows in the continental US. Geophys Res Lett 2001; 28: 2077–80. 10.1029/2000GL012745

[3] Kosaka Y, Xie SP. Recent global-warming hiatus tied to equatorial Pacific surface cooling. Nature 2013; 501: 403–7. 10.1038/nature12534 23995690

[4] Latif M, Barnett TP. Causes of decadal climate variability over the North Pacific and North American. Science 1994; 226: 634–7. 10.1126/science.266.5185.634 17793457

[5] Zhang R, Levitus S. Structure and cycle of decadal variability of upper-ocean temperature in the North Pacific. J Clim 1997; 10: 710–27. 10.1175/1520-0442(1997)010<0710:SACODV>2.0.CO;2

[6] Gu D, Philander SGH. Interdecadal climate fluctuations that depend on exchanges between the tropics and extratropics. Science 1997; 275: 805–7. 10.1126/science.275.5301.805 9012341

[7] Frankignoul C, Müller P, Zorita E. A simple model of the decadal response of the ocean to stochastic wind forcing. J Phys Oceanogr 1997; 27: 1533–46. 10.1175/1520-0485(1997)027<1533:ASMOTD>2.0.CO;2

[8] Qiu B . Kuroshio Extension variability and forcing of the Pacific decadal oscillations: responses and potential feedback. J Phys Oceanogr 2003; 33: 2465–82. 10.1175/2459.1

[9] Meehl GA, Hu AX, Castruccio F et al. Atlantic and Pacific tropics connected by mutually interactive decadal-timescale processes. Nat Geosci 2021; 14: 36–42. 10.1038/s41561-020-00669-x

[10] Zhang R, Delworth TL. Impact of the Atlantic Multidecadal Oscillation on North Pacific climate variability. Geophys Res Lett 2007; 34: 229–41. 10.1029/2007GL031601

[11] Wu S, Liu Z, Zhang R et al. On the observed relationship between the Pacific decadal oscillation and the Atlantic Multi-decadal Oscillation. J Oceanogr 2011; 67: 27–35. 10.1007/s10872-011-0003-x

[12] Nigam S, Sengupta A, Ruiz-Barradas A. Atlantic–Pacific links in observed multidecadal SST variability: is the Atlantic Multidecadal Oscillation's phase reversal orchestrated by the Pacific Decadal Oscillation? J Clim 2020; 33: 5479–505. 10.1175/JCLI-D-19-0880.1

[13] Wu B, Lin X, Yu L. North Pacific subtropical mode water is controlled by the Atlantic multi-decadal variability. Nat Clim Change 2020; 10: 238–43. 10.1038/s41558-020-0692-5

[14] Wu B, Lin X, Yu L. The decadal to multidecadal variability of mixed layer in the south of Kuroshio Extension region. J Clim 2020; 33: 7697–714. 10.1175/JCLI-D-20-0115.1

[15] McPhaden M, Zhang D. Slowdown of the meridional overturning circulation in the upper Pacific Ocean. Nature 2002; 415: 603–8. 10.1038/415603a 11832936

[16] Newman M, Alexander MA, Ault TR et al. The Pacific Decadal Oscillation, revisited. J Clim 2016; 29: 4399–427. 10.1175/JCLI-D-15-0508.1

[17] Lu P, McCreary JP Jr. Influence of the ITCZ on the flow of thermocline water from the subtropical to the equatorial Pacific Ocean. J Phys Oceanogr 1995; 25: 3076–88. 10.1175/1520-0485(1995)025<3076:IOTIOT>2.0.CO;2

[18] Alexander MA, Deser C. A mechanism for the recurrence of wintertime midlatitude SST anomalies. J Phys Oceanogr 1995; 25: 122–37. 10.1175/1520-0485(1995)025<0122:AMFTRO>2.0.CO;2

[19] Deser C, Alexander MA, Timlin MS. Understanding the persistence of sea surface temperature anomalies in midlatitudes. J Clim 2003; 16: 57–72. 10.1175/1520-0442(2003)016<0057:UTPOSS>2.0.CO;2

[20] Liu Z, Wu L. Atmospheric response to North Pacific SST: the role of ocean–atmosphere coupling. J Clim 2004; 17: 1859–82. 10.1175/1520-0442(2004)017<1859:ARTNPS>2.0.CO;2

[21] Masuzawa J . Subtropical mode water. Deep Sea Res 1969; 16: 463–72.

[22] Qu T, Chen J. A North Pacific decadal variability in subduction rate. Geophys Res Lett 2009; 36: L22602. 10.1029/2009GL040914

[23] Liu Q, Hu H. A subsurface pathway for low potential vorticity transport from the central North Pacific toward Taiwan Island. Geophys Res Lett 2007; 34: L12710. 10.1029/2007GL029510

[24] Wang ZY, Wen ZB, Hu HB et al. The characteristics of near-equatorial North Pacific low PV water and its possible influences on the equatorial subsurface ocean. J Geophys Res 2020; 125: e2020JC016282. 10.1029/2020JC016282

[25] Xie SP, Xu L, Liu Q et al. Dynamical role of mode water ventilation in decadal variability in the central subtropical gyre of the North Pacific. J Clim 2011; 24: 1212–25. 10.1175/2010JCLI3896.1

[26] Carton JA, Giese BS. A reanalysis of ocean climate using simple ocean data assimilation (SODA). Mon Weather Rev 2008; 136: 2999–3017. 10.1175/2007MWR1978.1

[27] Hurrell JW, Holland MM, Gent PR et al. The Community Earth System Model: a framework for collaborative research. Bull Am Meteorol Soc 2013; 94: 1339–60. 10.1175/BAMS-D-12-00121.1

[28] Fukumori I, Lee T, Cheng B et al. The origin, pathway, and destination of Niño-3 water estimated by a simulated passive tracer and its adjoint. J Phys Oceanogr 2004; 34: 582–604. 10.1175/2515.1

[29] Qu T, Gao S. Resurfacing of South Pacific tropical water in the Equatorial Pacific and its variability associated with ENSO. J Phys Oceanogr 2017; 47: 1095–106. 10.1175/JPO-D-16-0078.1

[30] Zhang Z, Sun X, Yang X. Understanding the interdecadal variability of East Asian summer monsoon precipitation: joint influence of three oceanic signals. J Clim 2018; 31: 5485–506. 10.1175/JCLI-D-17-0657.1

[31] Qiu B, Chen S, Schneider N et al. A coupled decadal prediction of the dynamic state of the Kuroshio Extension system. J Clim 2014; 27: 1751–64. 10.1175/JCLI-D-13-00318.1

[32] Fang J, Yang X. Structure and dynamics of decadal anomalies in the wintertime midlatitude North Pacific Ocean-atmosphere system. Clim Dyn 2016; 47: 1989–2007. 10.1007/s00382-015-2946-x

[33] Wang L, Yang X, Yang D et al. Two typical modes in the variabilities of wintertime North Pacific basin-scale oceanic fronts and associated atmospheric eddy-driven jet. Atmos Sci Lett 2017; 18: 373–80. 10.1002/asl.766

[34] Zhou G, Latif M, Greatbatch RJ et al. Atmospheric response to the North Pacific enabled by daily sea surface temperature variability. Geophys Res Lett 2015; 42: 7732–9. 10.1002/2015GL065356

[35] Ma X, Jing Z, Chang P et al. Western boundary currents regulated by interaction between ocean eddies and the atmosphere. Nature 2016; 535: 533–7. 10.1038/nature18640 27466126

[36] Tao L, Yang X, Fang J et al. PDO-related wintertime atmospheric anomalies over the midlatitude North Pacific: local versus remote SST forcing. J Clim 2020; 33: 6989–7010. 10.1175/JCLI-D-19-0143.1

[37] Schneider N, Miller AJ. Predicting western North Pacific Ocean climate. J Clim 2001; 14: 3997–4002. 10.1175/1520-0442(2001)014<3997:PWNPOC>2.0.CO;2

[38] Sugimoto S, Hanawa K. Remote reemergence areas of winter sea surface temperature anomalies in the North Pacific. Geophys Res Lett 2005; 32: L01606. 10.1029/2004GL021410

[39] Oka E, Qiu B. Progress of North Pacific mode water research in the past decade. J Oceanogr 2011; 68: 5–20. 10.1007/s10872-011-0032-5

[40] Minobe S . A 50–70 year climatic oscillation over the North Pacific and North America. Geophys Res Lett 1997; 24: 683–6. 10.1029/97GL00504

[41] Biondi F, Gershunov A, Cayan DR. North Pacific decadal climate variability since 1661. J Clim 2001; 14: 5–10. 10.1175/1520-0442(2001)014<0005:NPDCVS>2.0.CO;2

[42] Luo D, Chen Y, Dai A et al. Winter Eurasian cooling linked with the Atlantic multidecadal oscillation. Environ Res Lett 2017; 12: 125002. 10.1088/1748-9326/aa8de8

