# Supplementary material for: Re-emergence memory of subtropical mode-water links Atlantic and Pacific multidecadal variability
Source: Natl Sci Rev. 2025 Feb 14;12(5):nwaf047. doi: 10.1093/nsr/nwaf047 (PMC11970247; doi:10.1093/nsr/nwaf047)
Supplement: nwaf047_Supplemental_File [file nwaf047_supplemental_file.pdf]

Supplementary Data for

Reemergence Memory of Subtropical Mode Water Links Atlantic and Pacific Multidecadal Variability

Baolan Wu, Xiaopei Lin*, and Lisan Yu*

*Corresponding author. Email: linxiaop@ouc.edu.cn; lyu@whoi.edu

This PDF file includes:

Methods

Figs. S1 to S14

References for Supplementary Data

Methods

Observational and reanalysis products

Three observational data sets are used in this study to analyze the propagation pathway of the STMW. The first one is the Ishii dataset (version 7.3; [1]), which is monthly objective analysis of *in situ* observations (including expendable bathy-thermographs, conductivity-temperature-depth measurements from research ships and Argo floats) with a horizontal resolution of 1° × 1°. It covers the global ocean with 28 vertical levels from the sea surface to 3,000 m for the period from 1955 to the present. The other two observational data sets are the EN4 data (version 4.2.0-analyses-g10) produced by Met Office Hadley Center [2] and the one from Cheng [3] produced by Institute of Atmospheric Physics, Chinese Academy of Sciences (called IAP data in this study). These two data sets are processed in the same way as the Ishii data, all of which are monthly objective analyses of *in situ* observations with a horizontal resolution of 1° × 1°. The EN4 dataset provides the temperature and salinity fields with 42 depth levels from the sea surface down to 5,350 m starting from 1900. The IAP dataset has 41 vertical levels that extend to the 2,000 m depth below the sea surface for the period from 1940 onward. For more details, please refer to Ishii et al. [1], Good et al. [2] and Cheng et al. [3] for Ishii, EN4 and IAP data, respectively. The results are similar for all datasets and we present only the observational results from the Ishii data.

In addition, the ocean reanalysis dataset from the Simple Ocean Data Assimilation (SODA, version 2.2.4; [4]), which has horizontal resolution of 0.5° × 0.5° and a duration of 1871 to present is analyzed to support the findings. The SODA uses the Parallel Ocean Program physics ocean model and assimilates ocean observation data over 100 years. It has monthly

mean temporal resolution and 40 layers in the vertical direction, with the maximum depth over 5,000 m.

To examine the significance in the difference between the SST anomalies located in the subduction and those in the reemergence areas, four additional SST datasets are analyzed to further substantiate the findings based on the four aforementioned datasets (i.e., Ishii, EN4, IAP and SODA data). The four additions are the OISST (0.25°×0.25°) from the National Oceanic and Atmospheric Administration Optimum Interpolation SST Analysis version 2 (September 1981–December 2018; [5]), the HadISST from the MetOffice Hadley Centre (version 1; [6]), the Argo-based products RG09 made available by Roemmich and Gilson [7] that provides monthly temperature and salinity in a 1°×1° horizontal grid with 58 pressure levels to 2000 dbar, and the BOA from the Barnes objective analysis from China [8], which is monthly 1° gridded temperature and salinity dataset with 49 vertical levels from the surface to 1950 m depth.

The surface wind stress vectors and sea level pressure during 1955-2019 are from the National Center for Environmental Prediction (NCEP) and the National Center for Atmospheric Research (NCAR) global reanalysis [9]. It covers the global surface ocean with a T62 Gaussian grid. The SSALTO/DUACS [10] delayed-time multi-mission maps of the absolute dynamic topography is used in this study to characterize the climatological current field. The study used the sea surface height monthly data of 0.25°×0.25° spatial resolution from 1993 to 2019.

The AMO (sometimes also referred to as the Atlantic Multidecadal Variability) and PDO indices are used in this study, respectively [11,12]. The AMO index is based upon the average SST anomalies in the North Atlantic basin, typically over 0°N-65°N, 80°W-0°E.

The PDO index is defined as the leading principal component of the monthly SST anomalies in the North Pacific Ocean, poleward of 20°N.

Pre-industrial model simulation experiment

We use the pre-industrial model simulation experiment (hereafter PI-Control EXP.), based on the Community Earth System Model version 1.06 (CESM; [13]) of the National Center for Atmospheric Research, to demonstrate the robustness of the finding. The PI-Control EXP starts from a standard PI-Control EXP with Community Climate System Model, Version 4 (CCSM4), which has been running for 863 years. We run the CESM model with the same configuration for additional 600 years and use the last 200 years for our analysis. The PI-Control EXP. has spatial resolution of 1° × 1° and 60 levels in the vertical with the maximum depth over 5,000 m.

Pacemaker model experiment

The Pacemaker model experiment, similar to the PI-Control experiment, is conducted using the CESM v1.06 from the National Center for Atmospheric Research. The atmospheric component of this model is CAM4 with an F19 horizontal resolution (~2°), the same as used in the PI-Control experiment. The oceanic component is the Parallel Ocean Program version 2 (POP2), with a ~1° horizontal resolution. Basin-scale ocean mixed-layer temperature in the coupled model is restored as follows:

$$F = cH(T_r - T_m)/\tau, \quad (1)$$

where c is the heat content of sea water, H is the mixed layer depth, T_r is the restoring target temperature, T_m is the model temperature at each time step, and τ is the restoring time scale, which was set to 10 days in this study. External heating F was added to the

model to restore the basin-scale ocean mixed-layer temperature. The climate response to the restored ocean temperature was calculated as the difference between the Pacemaker model experiment and a control simulation. In the control simulation, the mixed-layer temperature was restored to the model climatology. In the perturbed run, an AMO-like SST warming pattern (derived by adding the SST trend from 1979-2014, when the AMO transitioned from a negative phase to a positive phase) over the Atlantic basin (Fig. S4 in Supplementary Data) was added to the mixed-layer temperature restored in the control run. We conducted 12-member ensemble simulations for 15 years using different initial conditions for both the control and perturbed runs.

Low-pass filtering and detrending data processing

To extract the dominant signal on decadal and multidecadal timescales, we apply a 7-year low-pass filtering and detrending to all time series.

Definition of the STMW

We define the STMW as a layer of vertical temperature gradient less than 1.5^{o}C (100 m)$^{-1}$ and calculate it from a linear fitting using the least squares method, by setting potential density in the range between 25.0 and 25.6 kg m^{-3}, and thickness greater than 50 m [14-17].

Lagrange-tracking method

To derive the propagation pathway of STMW along the thermocline, which is between 25.0 and 25.6 kg m^{-3} isopycnal surfaces (i.e., the STMW core density layer), we use the Lagrange-tracking method [18]. Specifically, the geostrophic current is firstly computed from the thermal wind equations using the temperature and salinity from the Ishii data. We

assume a level of no motion at 2000-m depth [19]. Secondly, we interpolate the current to the mean depth of the defined upper and lower density boundaries of the STMW (i.e., the mean depth between 25.0 and 25.6 kg m^{-3} isopycnal surfaces). Then we deploy tracers in the STMW formation and subduction area (28–35° N, 130° E–180°; [19]) on the mean depth. Finally, we calculate the propagation pathway of the STMW tracers by using the current along the circulation in each time step as shown in Fig. 1A and Fig. S1 in Supplementary Data. To better show propagation of the temperature anomalies, we divide the STMW trajectory into 12 sections with each section for about 1 year travelling time. Note that the climatological pathway of STMW (Fig. 1A) is computed using climatological geostrophic currents. This may underestimate the dispersion of the tracked particles induced by current variability and may also affect the estimate of the travelling time of the particles and STMW. Nonetheless, the uncertainty would not influence the conclusion of this study.

Passive tracer experiment

The tracing of STMW is investigated using a simulated passive tracer output from the Consortium for Estimating the Circulation and Climate of the Ocean (ECCO), which was developed from the framework of the Massachusetts Institute of Technology general circulation model [20]. The model is nearly global, extending from 80°S to 80°N, with a horizontal resolution of 1° globally, except within 20° of the equator, where meridional grid spacing is gradually reduced to 0.3° within 10° of the equator. Vertical resolution varies from 10 m within 150 m of the surface to 400 m near the bottom of the ocean. We use the offline tracing method [21,22] by treating the water mass with the same advection-diffusion equation as temperature and salinity in the ECCO model. The advantage of this

passive tracer experiment is that it includes the mixing during the tracking process. The initialized tracing STMW is selected between 160°-170°E, 27°-31°N (where the STMW could be advected outside of its formation region) in May 1993 (when the STMW formed in the winter time and subducted into the thermocline), with an arbitrary tracer unit value of 100%. We integrate forward in time using the velocity and mixing tensors of the model's offline outputs averaged at 10-day intervals from May 1993 to December 2016. A tracer-tagged water parcel is considered as reemergence when it reenters the mixed layer and no tracking of this water parcel will be further conducted. Two boundary layers are set, one along 15°N and the other along 170°W, to exclude the water parcels that transfer from outside of the study area.

Statistical significance test

The statistical significance of the linear regression coefficient and correlation between two-time series are based on a two-tailed Student's *t-test*. The degrees of freedom are estimated from the lag-1 autocorrelation following Bretherton et al. [23]:

$$N^* = N \cdot \frac{1-r_1 r_2}{1+r_1 r_2}, \quad (1)$$

where N^* is the effective sample size, N is the number of available time steps and r_1 and r_2 are the lag-1 autocorrelation of two autocorrelated time series.

Figs. S1 to S14

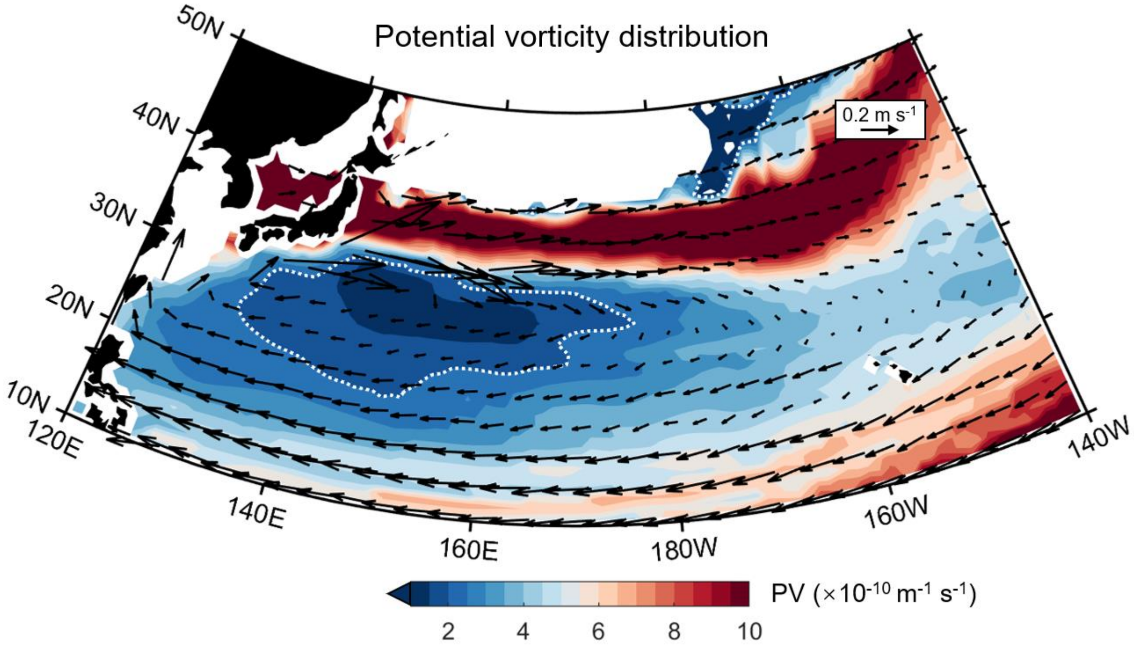

Fig. S1. Distribution of potential vorticity averaged over isopycnal surfaces between 25.0 and 25.6 kg m^{-3}. The shading denotes potential vorticity (unit in 10^{-10} m^{-1} s^{-1}), and the dashed white contour marks areas with low potential vorticity (< 2×10^{-10} m^{-1} s^{-1}) Climatological geostrophic velocity (vectors, unit in m s^{-1}) is calculated using the thermal wind equation, based on the Ishii temperature and salinity data.

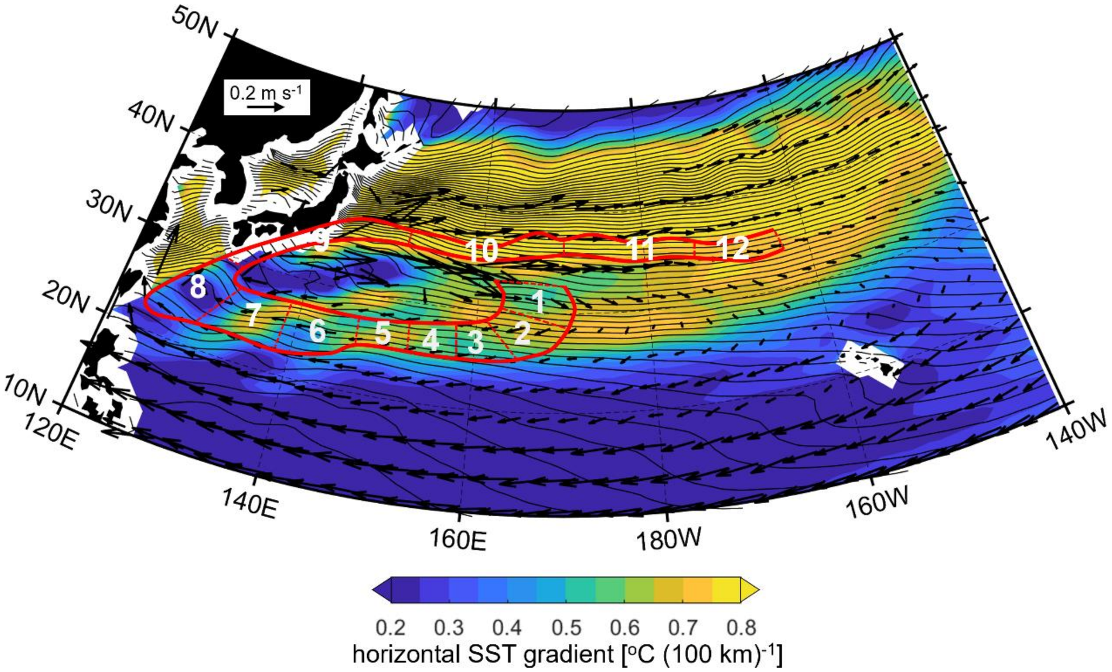

Fig. S2. The climatological current speed averaged over the isopycnal surfaces between 25.0 and 25.6 kg m^{-3}. The climatological velocity of geostrophic flow (unit in m s^{-1}) is computed from the thermal wind equation using the temperature and salinity from the Ishii data. Shading and contours denote the horizontal SST gradient [unit in ^{o}C (100 km)$^{-1}$] and SST (unit in ^{o}C), respectively. The positions of the 12 sections along the pathway are marked, with each section representing the travel time of about one year.

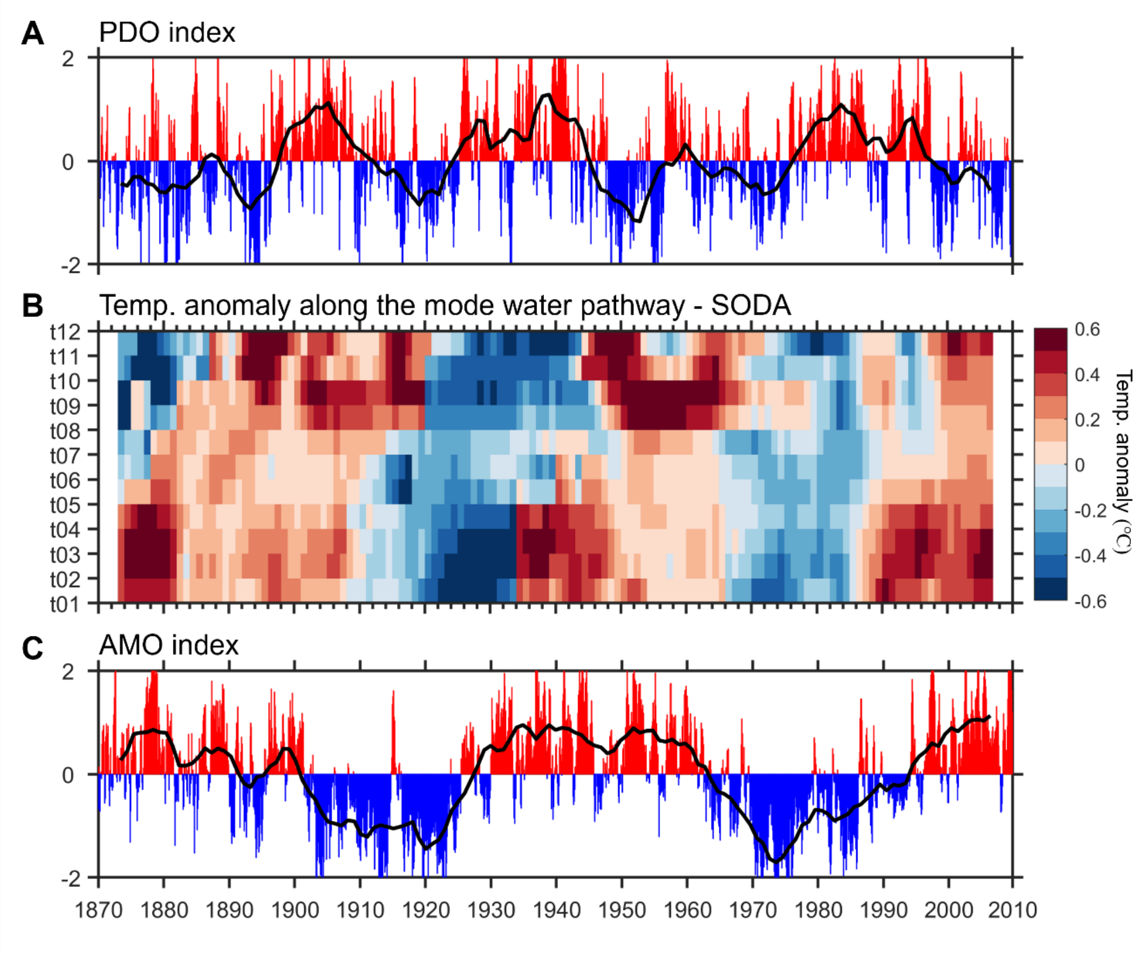

Fig. S3. Temperature anomalies propagate along the STMW pathway supported by SODA data. (A) The normalized monthly PDO index and its 7-year low-pass filtered time series (solid line). (B) The 7-year low-pass filtered temperature anomalies (unit in °C, derived from SODA data) averaged between 25.0 and 25.6 kg m-3 isopycnal surfaces along the 12 sections (i.e., the Y-axis t01~t12 denotes 1~12 sections shown in Figure 1A). (C) The normalized monthly AMO index and its 7-year low-pass filtered time series (solid line).

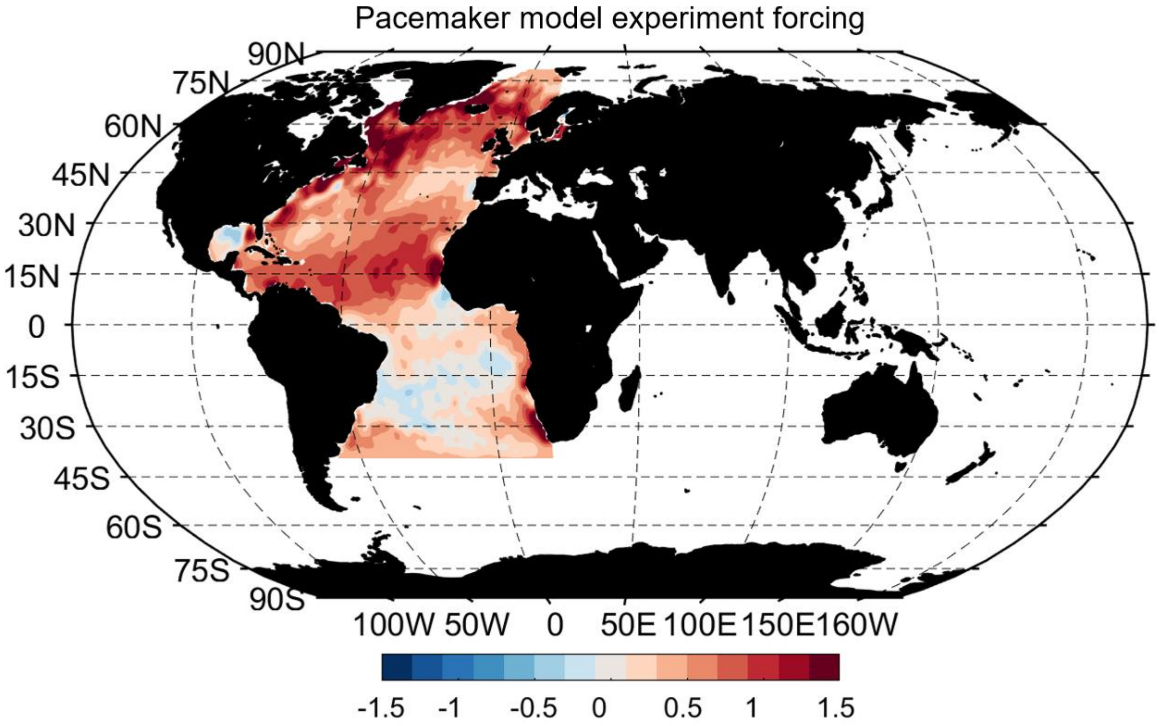

Fig. S4. Pacemaker model experiment forced by positive AMO SSTs. The observed SST trend (unit in ºC/36 yr) in the Atlantic Ocean from 1979-2014 was added to the modelled mixed layer temperature in the restoring forcing. In other regions, the mixed-layer temperature was restored to the model climatology. The climate response to the restored ocean temperature was calculated as the difference between the Pacemaker experiment and a control run experiment, in which the mixed-layer temperature was fully restored to the model climatology.

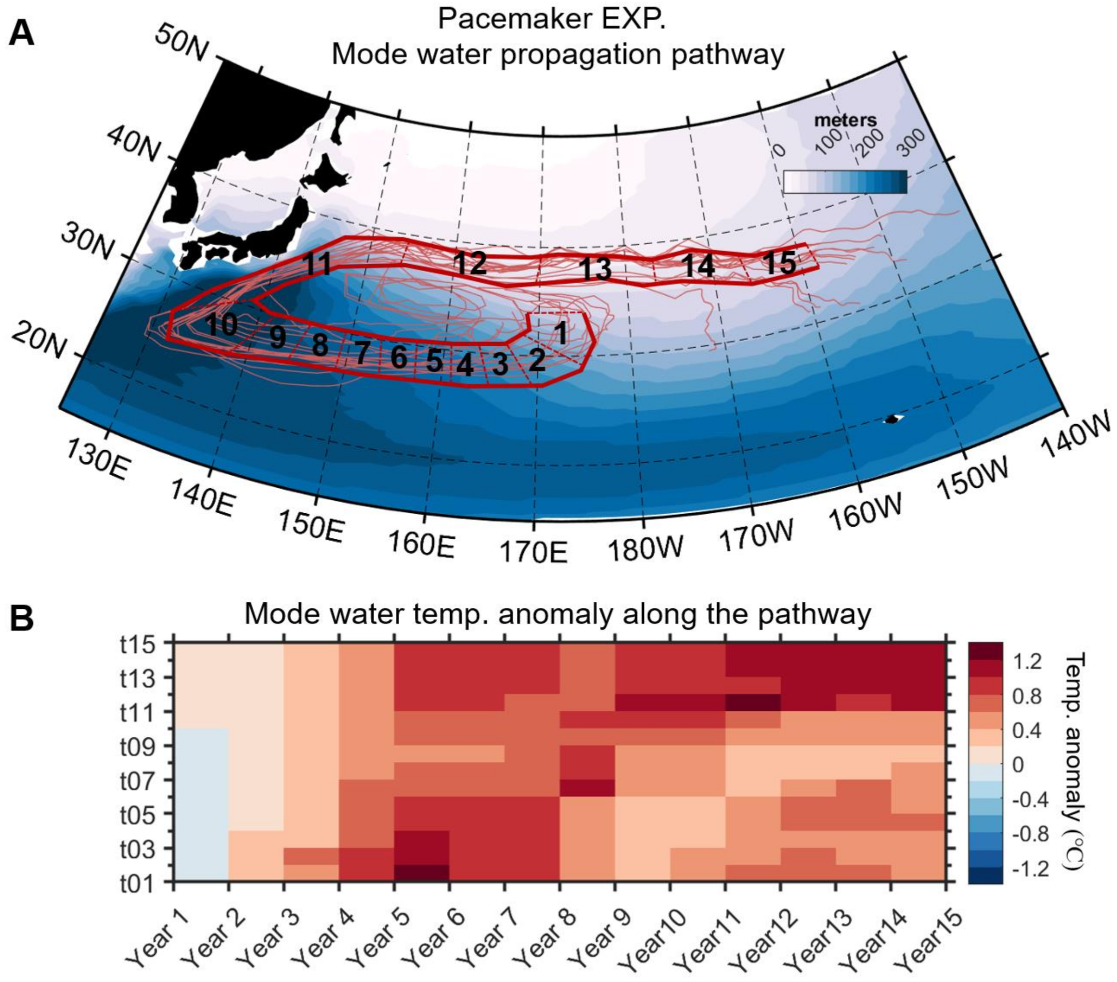

Fig. S5. Temperature anomalies propagating along the STMW pathway supported by the pacemaker model experiment. (A) The climatological pathway of STMW along the isopycnal surfaces between 25.0 and 25.6 kg m^{-3}, derived from the pacemaker model experiment (see Methods). Solid red lines represent the trajectories analyzed using a Lagrange-tracking method. Blue shading denotes the mean depth between 25.0 and 25.6 kg m^{-3} isopycnal surfaces (unit in m). The positions of 15 sections along the pathway are marked, with each section representing approximately one year of travel time. (B) Temperature anomalies (unit in ^{o}C) averaged over the 25.0 and 25.6 kg m^{-3} isopycnal surfaces along the 15 sections (denoted as t01~t15 on the Y-axis, corresponding to the 1~15 sections shown in Figure S5A).

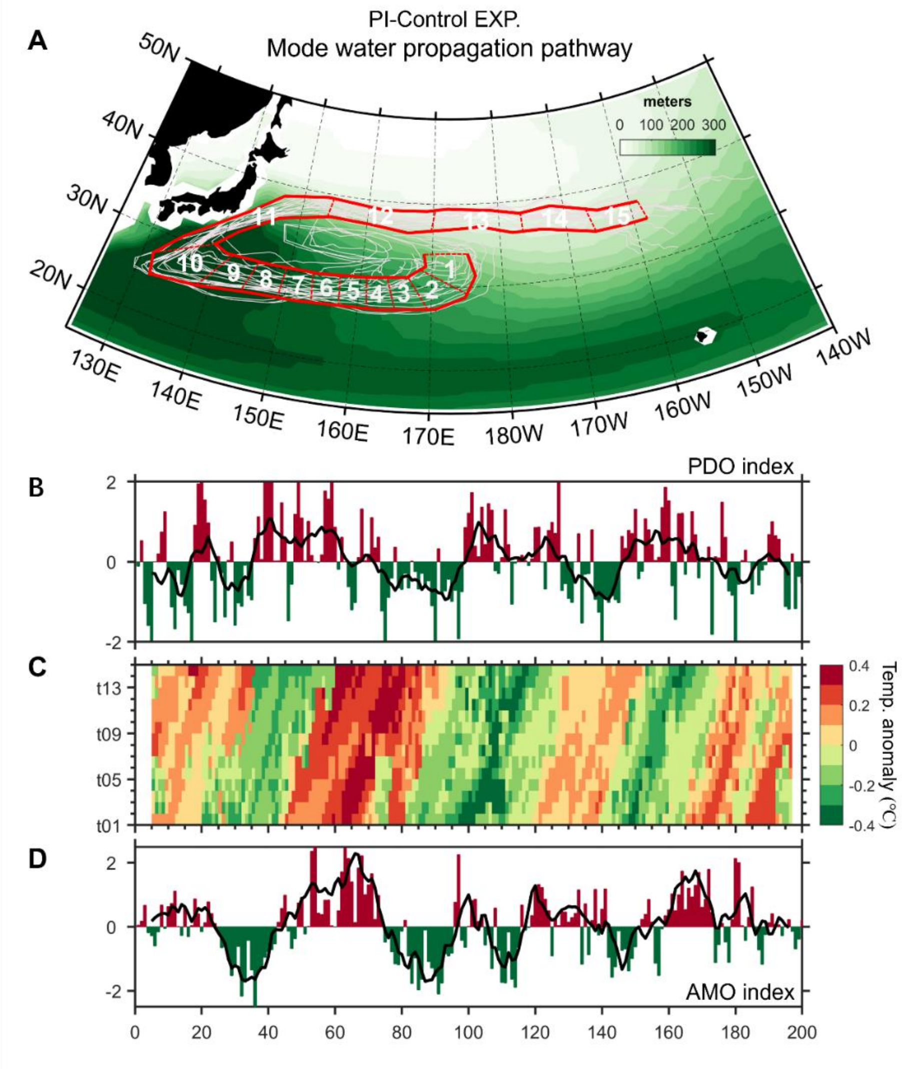

Fig. S6. Temperature anomalies propagate along the STMW pathway supported by the PI-Control EXP. (A) The climatological pathway of STMW for the isopycnal surfaces between 25.0 and 25.6 kg m^{-3}, derived from the PI-Control EXP. (see Methods). Solid white line represents the trajectories analyzed by a Lagrange-tracking method. Note that the trajectory is calculated by the STMW definition whose thickness is greater than 50 m. Green shading denotes the mean depth between 25.0 and 25.6 kg m^{-3} isopycnal surfaces (unit in m). The positions of the 15 sections along the pathway are marked, with each section representing the travel time of about one year. (B) The normalized monthly PDO index and its 7-year low-pass filtered time series (solid line). (C) The 7-year low-pass filtered temperature anomalies (unit in ºC) averaged between 25.0 and 25.6 kg m^{-3} isopycnal surfaces along the 15 sections (i.e., the Y-axis t01~t15 denotes 1~15 sections shown in Figure S3A). (D) The normalized monthly AMO index and its 7-year low-pass filtered time series (solid line).

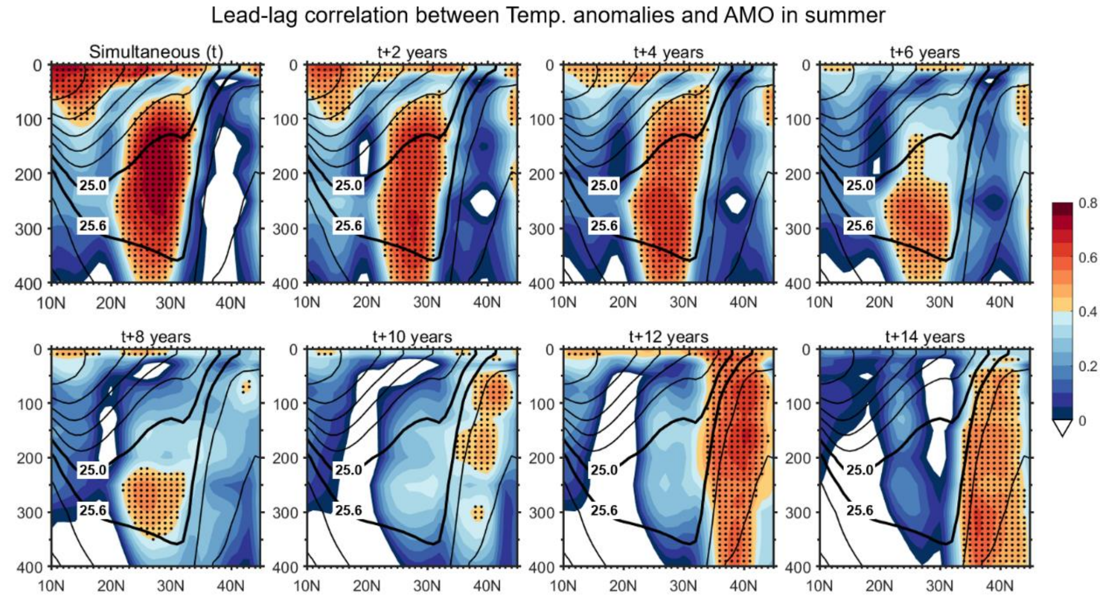

Fig. S7. Vertical propagation of the temperature anomalies in summer. The lead-lag correlation between temperature anomalies (averaged between 140°E and 170°E transect, derived from the Ishii data) and the AMO index in summer, both smoothed using a 7-year low-pass filter, is shown at 2-yr intervals (shading, unit in ^{o}C). Contours indicate potential density with 0.6 kg m^{-3} intervals. Thick solid contours denote potential densities of 25.0 and 25.6 kg m^{-3}, which define the upper and lower density boundaries of the STMW. Dot-shaded areas indicate correlations significant at the 95% confidence interval. A 7-year low-pass filter was applied to each season.

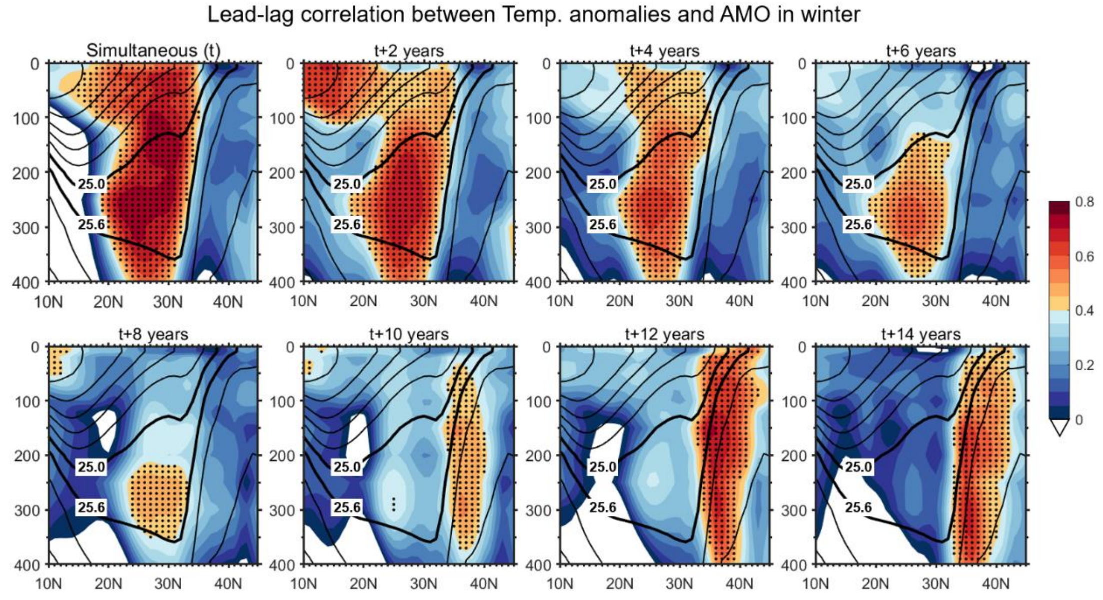

Fig. S8. Vertical propagation of the temperature anomalies in winter. The lead-lag correlation between temperature anomalies (averaged between 140°E and 170°E transect, derived from the Ishii data) and the AMO index in winter, both smoothed using a 7-year low-pass filter, is shown at 2-yr intervals (shading, unit in ^{o}C). Contours indicate potential density with 0.6 kg m^{-3} intervals. Thick solid contours indicate potential densities of 25.0 and 25.6 kg m^{-3}, which define the upper and lower density boundaries of the STMW. Dot-shaded areas represent the correlations significant at the 95% confidence interval. A 7-year low-pass filter was applied to each season.

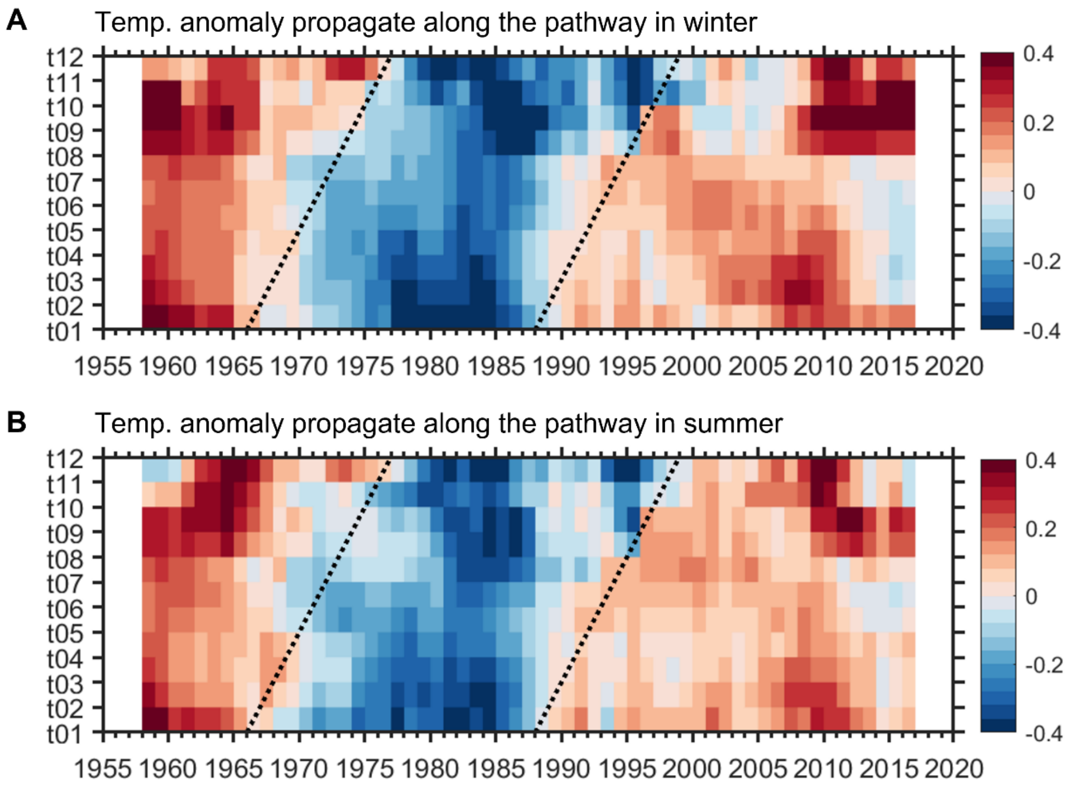

Fig. S9. Nonseasonal temperature anomalies along the STMW pathway. (A) The 7-year low-pass filtered temperature anomalies (unit in °C, derived from the Ishii data) averaged between 25.0 and 25.6 kg m^{-3} isopycnal surfaces along the 12 sections in winter. (B) Same as (A), but in summer.

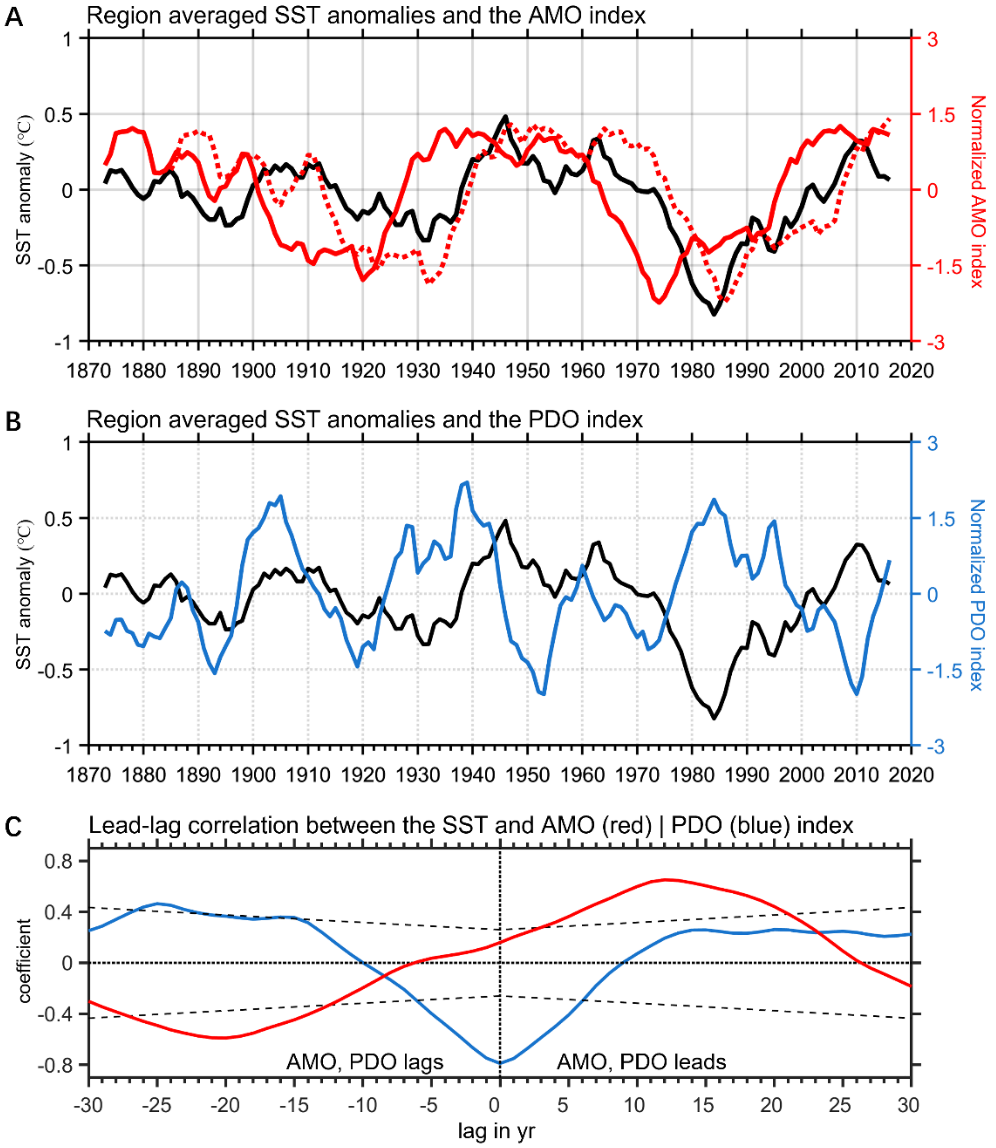

Fig. S10. Time series of region averaged SST anomalies, AMO and PDO index. (A) Time series of the region averaged (150°E-160°W, 35°-42°N) SST anomalies (black line) and normalized AMO index (red solid line) smoothed by a 7-year low-pass filter. Red dashed line indicates the AMO index with 12 year lags. (B) Time series of the region averaged (150°E-160°W, 35°-42°N) SST anomalies (black line) and normalized PDO index (blue line) smoothed by a 7-year low-pass filter. (C) Lead-lag correlation between the SST (averaged in the region of 150°E-160°W, 35°-42°N) and AMO index (red line), PDO index (blue line), all smoothed by a 7-year low-pass filter. Dashed lines indicate 5% significance.

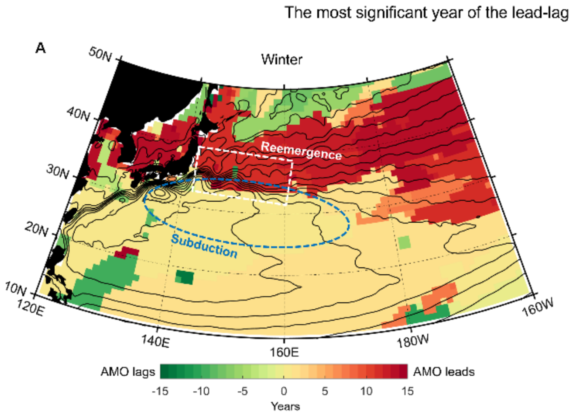

Fig. S11. The SST anomalies related to AMO in winter and summer. (A) The most significant year in the lead-lag correlation between SST and AMO index for the winter season, both smoothed by a 7-year low-pass filter (shading). Contours represent the absolute dynamic topography derived from AVISO, with interval of 10 cm. The white box and blue oval shapes denote the climatological reemergence area and subduction area, respectively. (B) Same as (A), but for the summer season.

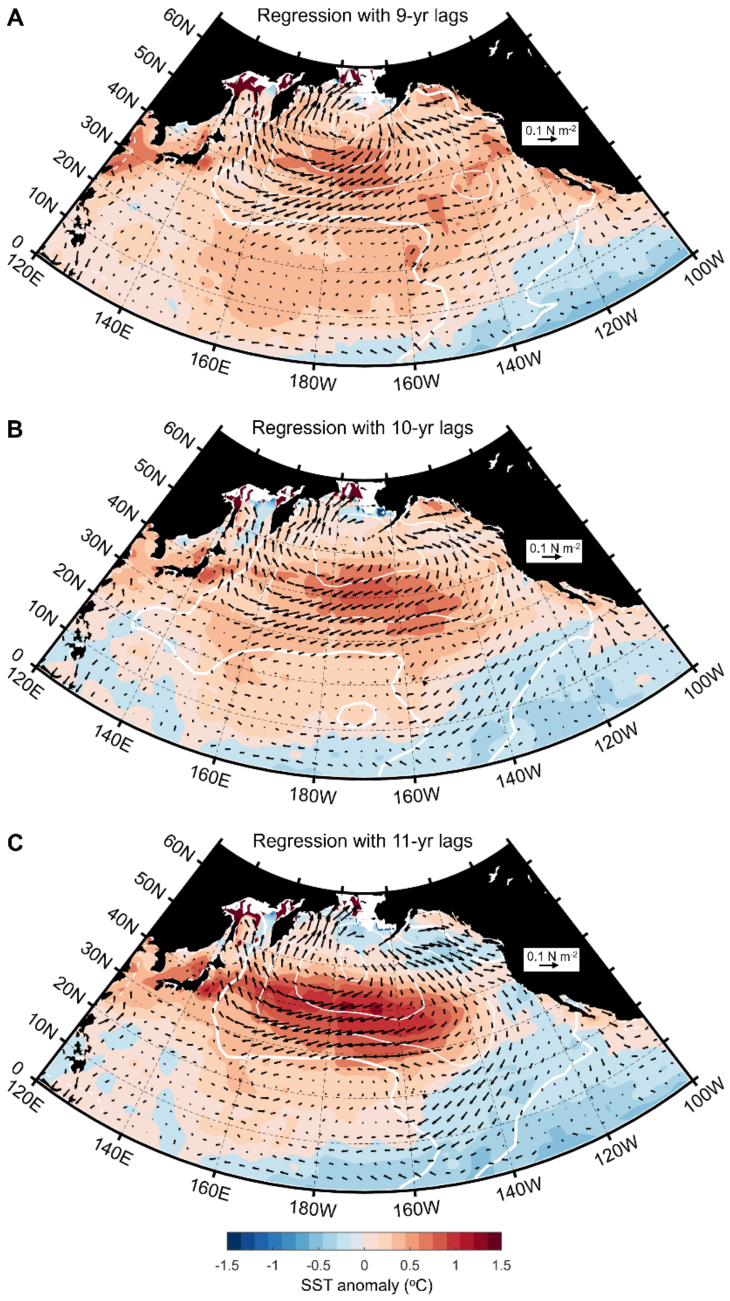

Fig. S12. Sequence of time-lag regression maps show how the reemergence of the AMO-induced SST anomalies could trigger a PDO. The spatial structure of the time-lag regression of SST (shading, unit in ºC), wind stress (vector, unit in N/m^2) and sea level pressure (white contours, unit in hPa) on the AMO index at lag (A) 9 years, (B) 10 years and (C) 11 years. Contour intervals are 1.2 hPa. Positive (negative) contours are dashed (solid). Thick solid contours indicate sea level pressure of 0.

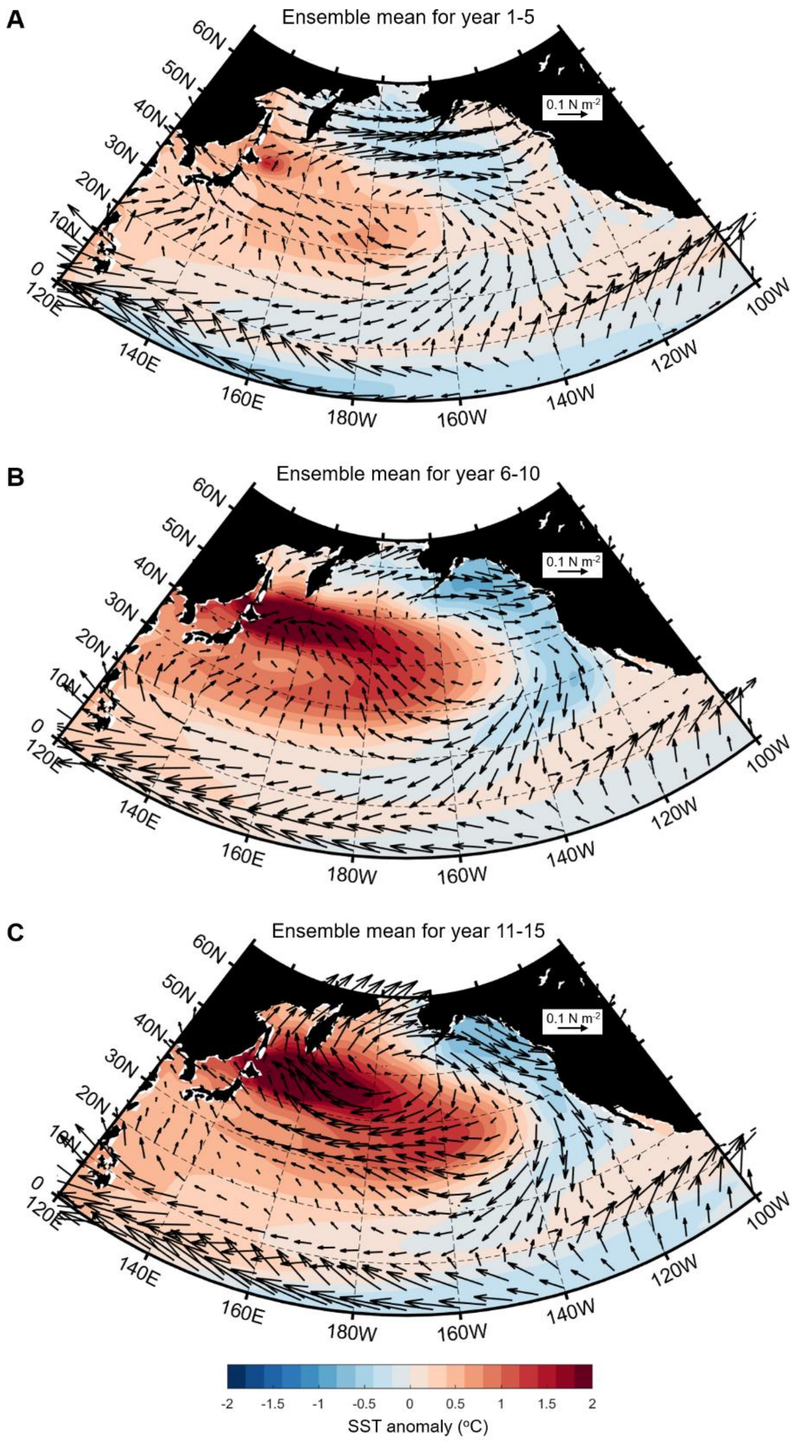

Fig. S13. Ensemble mean maps showing the evolution of the AMO-induced PDO pattern over time. Five-year averaged SST anomalies (shading, unit in ^{o}C) and wind stress (vectors, unit in N/m^2) for (A) years 1-5, (B) years 6-10 and (C) years 11-15from the pacemaker model experiment.

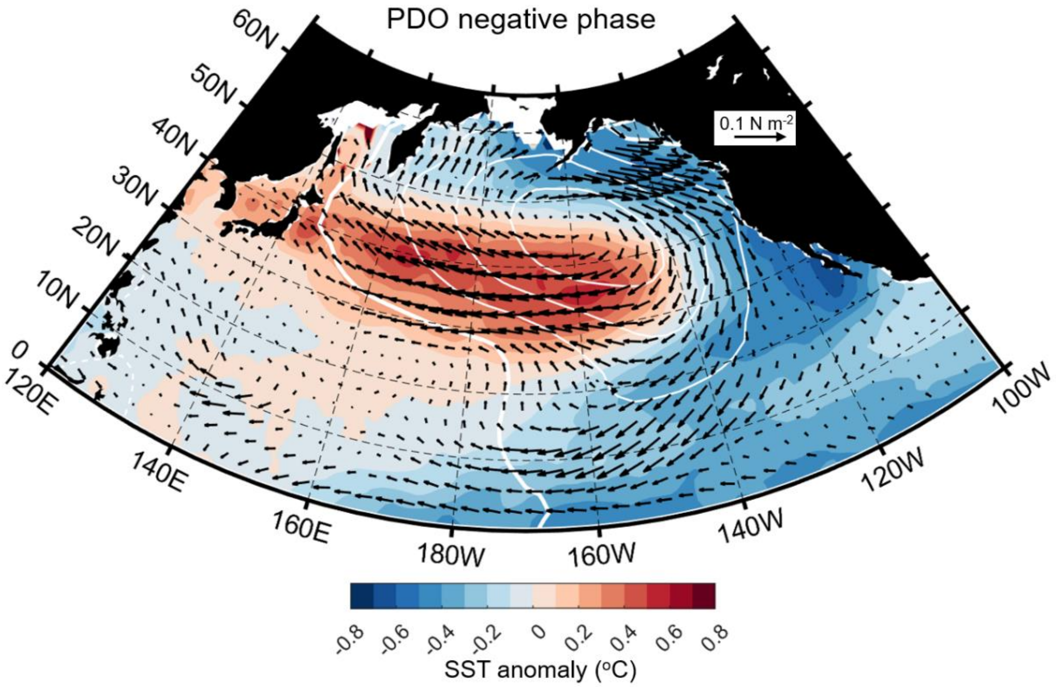

Fig. S14. Spatial pattern of PDO negative phase. The spatial structure of the SST (color shading, unit in °C), wind stress (vector, unit in N/m^2) and sea level pressure (white contours, unit in hPa) of PDO negative phase. Contour intervals are 0.4 hPa, with positive contours as dashed lines and negative contours as solid lines. The thick white contour marks the 0-hPa sea level pressure.

References for Supplementary Data

1. Ishii M, Fukuda Y and Hirahara *et al.* Accuracy of Global Upper Ocean Heat Content Estimation Expected from Present Observational Data Sets. *SOLA* 2017; **13**: 163-167.
2. Good SA, Martin MJ and Rayner NA. EN4: quality controlled ocean temperature and salinity profiles and monthly objective analyses with uncertainty estimates. *J Geophys Res* 2013; **118**: 6704-6716.
3. Cheng L, Trenberth K and Fasullo J *et al.* Improved estimates of ocean heat content from 1960–2015. *Sci Adv* 2017; **3**: e1601545.
4. Carton JA and Giese BS. A reanalysis of ocean climate using simple ocean data assimilation (SODA). *Monthly Weather Review* 2008; **136**: 2999-3017.
5. Reynolds RW, Smith TM and Liu C *et al.* Daily high-resolution-blended analyses for sea surface temperature. *J Clim* 2007; **20**: 5473–5496.
6. Rayner NA, Parker DE and Horton EB *et al.* Global analyses of sea surface temperature, sea ice, and night marine air temperature since the late nineteenth century. *J Geophys Res* 2003; **108**: D14.
7. Roemmich D and Gilson J. The 2004-2008 mean and annual cycle of temperature, salinity, and steric height in the global ocean from the Argo Program. *Progress in Oceanogr* 2009; **82**: 81–100.
8. Li H, Xu F and Zhou W*et al.* Development of a global gridded Argo data set with Barnes successive corrections. *J Geophys Res* 2017; **122**: 866-889.
9. Kalnay E, Kanamitsu M and Kistler R *et al.* The NCEP/NCAR 40-year reanalysis project. *Bull Am Meteorol Soc* 1996; **77**: 437-472.
10. SALTO/DUACS (2013) SSALTO/DUACS User Handbook: (M)SLA and (M)ADT Near-real time and delayed time products
11. Enfield DB, Mestas-Nunez AM and Trimble PJ. The Atlantic multidecadal oscillation and its relation to rainfall and river flows in the continental US. *Geophys Res Lett* 2001; **28**: 2077–2080.
12. Mantua NJ, Hare SR and Zhang Y *et al.* A Pacific interdecadal climate oscillation with impacts on salmon production. *Bull Am Meteorol Soc* 1997; **78**: 1069-1079.
13. Hurrell JW, Holland MM and Gent PR *et al.* The Community Earth System Model: A framework for collaborative research. *Bull Am Meteorol Soc* 2013; **94**: 1339-1360.
14. Masuzawa J. Subtropical Mode Water. *Deep Sea Res* 1969; **16**: 463-472.
15. Qu T and Chen J. A North Pacific decadal variability in subduction rate. *Geophys Res Lett* 2009; **36**: L22602.
16. Hanawa K and Talley LD. “Mode waters” in *Ocean Circulation and Climate*, G. Siedler, J. Church, J. Gould, Eds., vol. 77. (Academic Press, San Diego, CA, pp. 373-386, 2001).

17. Wang R, Yu F and Nan F. Weakening of subduction in the subtropical mode water formation region observed during 2003–2013. *J Geophys Res* 2015; **120**: 7271-7281.

18. Liu Q and Hu H. A subsurface pathway for low potential vorticity transport from the central North Pacific toward Taiwan Island. *Geophys Res Lett* 2007; **34**: L12710.

19. Wu B, Lin X and Yu L. Poleward Shift of the Kuroshio Extension Front and its Impact on the North Pacific Subtropical Mode Water in the recent decades. *J Phys Oceanogr* 2021; **51**: 457-474.

20. Marshall J, Adcroft A and Hill C *et al.* A finite-volume, incompressible Navier Stokes model for studies of the ocean on parallel computers. *J Geophys Res* 1997; **102**: 5753–5766.

21. Fukumori I, Lee T and Cheng B *et al.* The origin, pathway, and destination of Niño-3 water estimated by a simulated passive tracer and its adjoint. *J Phys Oceanogr* 2004; **34**: 582–604.

22. Qu T and Gao S. Resurfacing of South Pacific Tropical Water in the Equatorial Pacific and Its Variability Associated with ENSO. *J Phys Oceanogr* 2017; **47**: 1095-1106.

23. Bretherton CS, Widmann M and Dymnidov VP *et al.* The effective number of spatial degrees of freedom of a time-varying field. *J Clim* 1999; **12**: 1990–2009.
